# Maxillary Arch Morphology in Unilateral Buccally and Palatally Impacted Maxillary Canines: A Three-Dimensional Digital Model Study

**DOI:** 10.3390/diagnostics16131971

**Published:** 2026-06-24

**Authors:** Nuri Can Tanrısever, Özge Nur Kartal, Ayşegül Dilara Güvenç Tokur, Mehmet Okan Akçam

**Affiliations:** Department of Orthodontics, School of Dentistry, University of Ankara, Ankara 06560, Türkiye

**Keywords:** cone-beam computed tomography, dental arch, imaging, three-dimensional, maxilla, tooth, canine, tooth, impacted

## Abstract

**Background/Objectives:** Impacted maxillary canines are frequently associated with variations in maxillary arch morphology; however, the relationship between impaction position and three-dimensional arch characteristics remains unclear. This study aimed to evaluate the association between buccally and palatally impacted maxillary canines and maxillary arch morphology using CBCT and three-dimensional digital model analysis. **Methods:** This retrospective cross-sectional study included CBCT images and three-dimensional dental models of 86 individuals with unilateral impacted maxillary canines (mean age: 16.1 ± 0.72 years). Impacted canines were classified as buccal or palatal according to CBCT findings. Maxillary arch morphology was assessed using digital model analysis. Statistical comparisons between groups were performed using independent-samples *t*-tests (*p* < 0.05). **Results:** The buccally impacted group demonstrated significantly greater arch length, higher arch length-to-arch width ratios, greater mesiodistal width of the four maxillary incisors and increased tooth–arch discrepancy (*p* < 0.05). In contrast, intermolar width and available arch space were significantly greater in the palatally impacted group (*p* < 0.05). No significant differences were identified in arch width or palatal depth measurements between groups (*p* > 0.05). Intra-examiner reliability demonstrated excellent agreement (ICC > 0.90). **Conclusions:** Maxillary dental arch morphology differed according to the position of impacted maxillary canines. Buccal impaction was associated with sagittal arch elongation and increased tooth–arch discrepancy. In contrast, palatal impaction was not consistently associated with reduced transverse dental arch dimensions within the measurements evaluated in this study. These findings contribute to a better understanding of the association between impacted canine position and maxillary dental arch morphology and may assist clinicians in the morphological assessment of patients with impacted maxillary canines.

## 1. Introduction

Tooth eruption is a physiological developmental process through which teeth migrate from their intra-alveolar position into the functional occlusal plane [1]. Disturbances during this process may result in delayed eruption or tooth impaction, which has been defined as delayed or failed eruption following root development or the absence of eruption after the expected eruption period [2,3]. Among all permanent teeth, maxillary canines are the second most frequently impacted teeth after third molars [4]. Compared with other maxillary teeth, maxillary canines exhibit a prolonged developmental period, originate from a deeper odontogenic position, and follow a more complex eruption pathway before reaching the occlusal plane [5,6].

The reported prevalence of impacted maxillary canines varies considerably across populations and study methodologies. Earlier epidemiological studies reported prevalence rates ranging from approximately 0.3% to 2.4% [7], whereas more recent investigations have demonstrated a wider range, from approximately 0.97% to 7.10%, depending on population characteristics, diagnostic criteria, and imaging methods [8,9]. Impacted maxillary canines are more frequently observed in Caucasian populations and occur approximately three times more often in females than in males [10,11]. Previous studies have demonstrated that impacted maxillary canines may deviate from their normal eruption pathway and become impacted either buccally or palatally, with palatal impaction reported nearly three times more frequently than buccal impaction [12,13].

With regard to etiology, palatal impaction has traditionally been associated with genetic predisposition and dental developmental anomalies, whereas buccal impaction has been linked more closely to space deficiency and variations in dental arch morphology [11,14]. In patients with palatally impacted canines, adequate or even excessive arch space has frequently been reported, whereas buccally impacted canines are commonly associated with dental crowding [15,16,17,18].

However, recent evidence has challenged this traditional concept by suggesting that palatal impaction may also be associated with maxillary transverse deficiency and altered skeletal morphology [19,20]. These findings indicate that skeletal and dentoalveolar characteristics may play a more substantial role in the etiology of canine impaction than previously assumed.

Despite numerous investigations evaluating impacted maxillary canines, the relationship between impaction position and maxillary arch morphology remains controversial. Some studies have reported reduced transverse dimensions, altered palatal morphology, and narrower maxillary arches in patients with palatally impacted canines, suggesting a potential association between canine impaction and maxillary morphological characteristics [21,22]. Conversely, systematic reviews and other investigations have highlighted inconsistent findings and considerable heterogeneity among studies, with no consensus regarding the presence or extent of maxillary morphological differences associated with canine impaction [23]. These inconsistent findings may partly be explained by methodological limitations of previous studies, many of which relied on two-dimensional radiographic techniques or conventional plaster model measurements that may not accurately reflect the complex three-dimensional anatomy of the maxillary arch. Understanding the relationship between maxillary arch morphology and canine impaction may improve early diagnosis, prediction of eruption disturbances, and interceptive orthodontic treatment planning.

Recent advances in three-dimensional imaging and digital model analysis have enabled more accurate evaluation of dentoalveolar morphology compared with conventional two-dimensional approaches. Cone-beam computed tomography (CBCT) and digital dental model analysis allow comprehensive assessment of maxillary arch morphology, including arch width, arch length, palatal depth, and transverse dimensions, with greater accuracy and reproducibility compared with conventional methods. Several recent three-dimensional studies have investigated maxillary arch morphology in patients with buccally and palatally impacted maxillary canines, evaluating parameters such as arch dimensions, palatal morphology, arch form, and available arch space [24,25]. However, direct comparisons between unilateral buccally and palatally impacted maxillary canines remain relatively limited, and the reported findings have been inconsistent. Furthermore, few studies have simultaneously evaluated arch morphology, palatal morphology, and tooth–arch size relationships using standardized three-dimensional digital model analysis.

Although previous studies have investigated selected aspects of maxillary morphology associated with impacted canines, most focused on isolated morphological parameters or included heterogeneous samples. In addition, studies directly comparing unilateral buccally and palatally impacted maxillary canines using standardized three-dimensional digital model analysis remain limited, particularly in balanced study groups with equal representation of buccal and palatal impactions. Consequently, the influence of impaction position on the combined characteristics of arch morphology, palatal morphology, and tooth–arch size relationships has not yet been fully clarified. Beyond the evaluation of individual morphometric variables, the present study sought to characterize the broader pattern of maxillary arch morphology associated with different impaction positions through the combined assessment of arch morphology, palatal morphology, and tooth–arch size relationships.

Therefore, this study aimed to compare maxillary arch morphology in patients with unilateral buccally and palatally impacted maxillary canines using three-dimensional digital model analysis, with impacted canine position determined by CBCT imaging.

## 2. Materials and Methods

### 2.1. Study Design and Sample

This retrospective cross-sectional study consisted of cone-beam computed tomography (CBCT) images and three-dimensional (3D) dental models obtained from 86 individuals with unilateral impacted maxillary canines who presented to the Department of Orthodontics, Faculty of Dentistry, Ankara University (mean age: 16.10 ± 0.72 years; 42 females, 44 males). All CBCT images were acquired using the same CBCT unit (Carestream CS 8100 3D, Carestream Health Inc., Rochester, NY, USA), and all three-dimensional dental models were obtained using the same intraoral scanner (3Shape TRIOS^®^ 3 Intraoral Scanner, 3Shape A/S, Copenhagen, Denmark).

This study was conducted in accordance with the principles of the Declaration of Helsinki and approved by the Ankara University Non-Clinical Scientific Research Ethics Committee (Meeting No: 19; Decision Date: 3 March 2026). Due to the retrospective design of the study and the use of anonymized archived records, the requirement for informed consent was waived by the ethics committee.

The diagnosis of impacted maxillary canines was established using clinical and radiographic records, including intraoral photographs and panoramic radiographs. Teeth that failed to erupt into the dental arch despite a minimum of six months having elapsed beyond the expected eruption age and that demonstrated at least three-quarters of root development on panoramic radiographs were classified as impacted. Age was calculated as the interval between the date of birth and the date of CBCT acquisition and was expressed in decimal years. The age of the included individuals ranged from 14.42 to 17.42 years (mean age: 16.10 ± 0.72 years), representing the age interval in which delayed eruption of the maxillary canine could be reliably assessed using the predefined clinical and radiographic criteria. Initially, 104 individuals with impacted maxillary canines were screened. Individuals presenting with craniofacial anomalies, congenital tooth agenesis, previous orthodontic treatment, poor-quality CBCT images or digital models, or multiple impacted teeth other than third molars were excluded from the study. Third molars were not considered when determining the presence of multiple impacted teeth because their eruption and root development may still be ongoing during adolescence.

**Figure 1:**
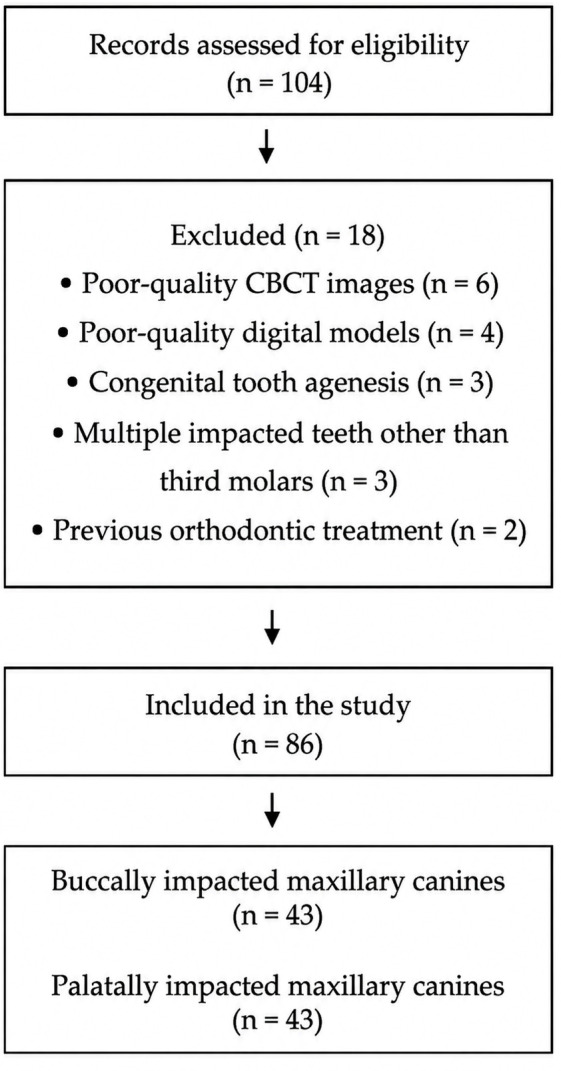
Flow diagram illustrating participant selection and allocation to the buccally and palatally impacted canine groups.

Following application of the exclusion criteria, 86 individuals were included in the final study sample (Figure 1). Only individuals with unilateral impacted maxillary canines were included in the study. Because the primary objective of the study was to compare maxillary arch morphology between buccally and palatally impacted canines, equal numbers of individuals were included in each group to facilitate balanced statistical comparisons and minimize the potential influence of unequal group sizes on the study findings.

CBCT images were used exclusively for the localization and classification of impacted maxillary canines as buccal or palatal. All morphometric assessments of maxillary arch morphology were performed on three-dimensional digital dental models obtained from intraoral scans.

### 2.2. Classification of Impacted Canines

**Figure 2:**
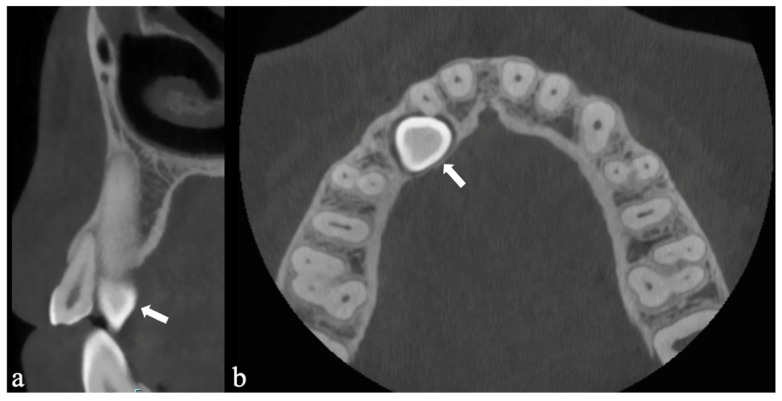
Classification of impacted maxillary canines according to CBCT images. (**a**) Sagittal section demonstrating the relationship between the impacted canine and the lateral incisor root. (**b**) Axial section showing the buccopalatal localization of the impacted canine. Arrows indicate the position of the impacted canine.

Impacted maxillary canines were classified according to their position on CBCT images by evaluating their location on axial sections and their relationship to the root of the lateral incisor on sagittal sections. Canines located close to the buccal cortical plate and positioned buccal to the lateral incisor root were classified as buccally impacted, whereas canines located close to the palatal cortical plate and positioned palatal to the lateral incisor root were classified as palatally impacted (Figure 2).

### 2.3. Three-Dimensional Dental Model Analysis

**Figure 3:**
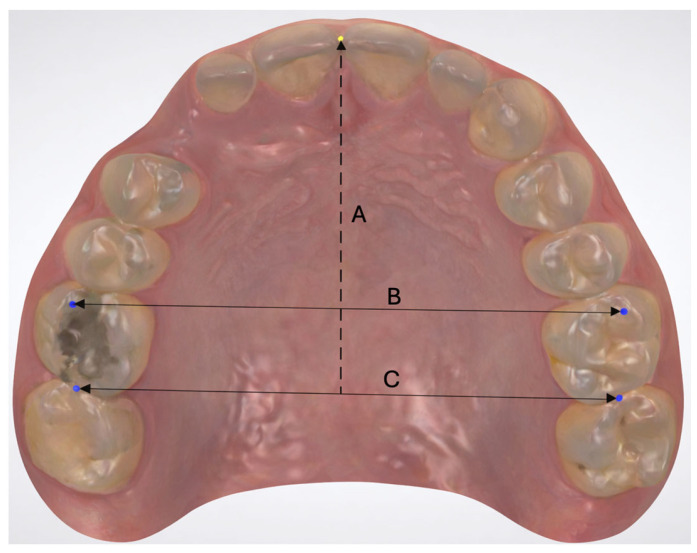
Three-dimensional maxillary dental model used for evaluation of arch morphology. A: arch length measured from the incisal reference point to the intermolar reference line; B: arch width measured between the mesiobuccal cusp tips of the maxillary first molars; C: reference line passing through the distal contact points of the maxillary first molars.

To evaluate maxillary dental arch morphology in the buccal and palatal impaction groups, the ratio of arch length to arch width (arch length/arch width × 100) was calculated. Arch length was defined as the perpendicular distance from the incisal edge of the maxillary central incisors to a line passing through the distal contact points of the first molars. In cases where sagittal positioning of the central incisors differed because of crowding or rotation, the most anteriorly positioned central incisor was used as the reference point. Arch width was defined as the distance between the mesiobuccal cusp tips of the maxillary first molars (Figure 3).

**Figure 4:**
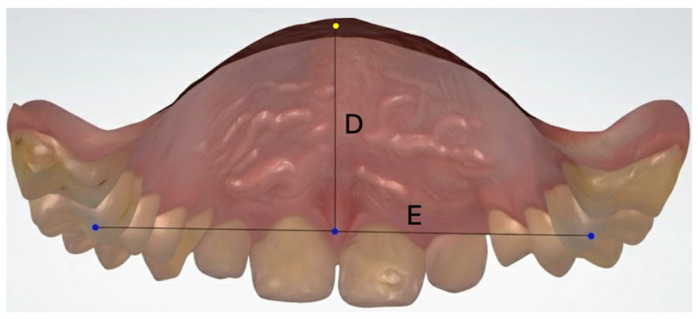
Three-dimensional maxillary dental model used for evaluation of palatal vault morphology. D: palatal vault depth measured from the deepest point of the palatal vault to the intermolar reference line; E: intermolar reference line passing through the mesiopalatal cusp tips of the maxillary first molars.

To assess palatal vault morphology, the ratio of palatal vault depth to intermolar width (palatal vault depth/intermolar width × 100) was calculated. Palatal vault depth was defined as the perpendicular distance from the deepest point of the palatal vault to a line passing through the mesiopalatal cusp tips of the maxillary first molars (Figure 4). Intermolar width was measured as the distance between the mesiopalatal cusp tips of the maxillary first molars.

**Figure 5:**
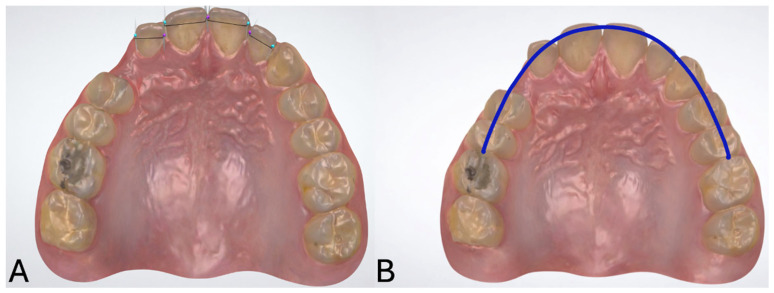
Three-dimensional dental models used for evaluation of eruption space for the maxillary canines. (**A**) Measurement of the total mesiodistal widths of the four maxillary incisors. (**B**) Curvilinear measurement of the available arch space between the mesial contact points of the maxillary first molars.

To evaluate the eruption space available for the canines, the ratio of the total mesiodistal widths of the four maxillary incisors to the available arch space (×100) was calculated. Available arch space was defined as the curvilinear distance between the mesial contact points of the maxillary first molars (Figure 5).

Before measurement procedures, all three-dimensional dental models were oriented using a standardized reference plane based on the maxillary occlusal plane, defined by the occlusal surfaces of the posterior teeth and the incisal edges of the anterior teeth, to ensure consistent spatial positioning and improve measurement reproducibility. Anatomical landmarks used for the measurements were identified directly on the digital models according to the predefined measurement definitions described above. All measurements were performed on three-dimensional maxillary dental models by a single calibrated investigator (Ö.N.K.), an orthodontist with experience in three-dimensional digital model analysis and orthodontic research, using OrthoAnalyzer software (OrthoAnalyzer™ 2019, 3Shape A/S, Denmark).

### 2.4. Statistical Analysis

Statistical analyses were performed using SPSS software (version 26.0; IBM Corp., Armonk, NY, USA). Descriptive statistics were calculated to summarize the demographic characteristics of the study population. Because the sample size exceeded 50 participants (*N* = 86), normality of the data distribution was assessed using skewness and kurtosis statistics. Values between −2 and +2 were considered indicative of a normal distribution. All variables demonstrated skewness and kurtosis values within these limits. Homogeneity of variances was assessed using Levene’s test prior to performing independent-samples *t*-tests.

A priori power analysis was performed using G Power software (version 3.1; Heinrich Heine University, Düsseldorf, Germany) before data collection to determine the minimum required sample size. Based on an independent-samples *t*-test, with an effect size of 0.80, representing a large effect according to Cohen’s criteria, a significance level (α) of 0.05, and a statistical power (1−β) of 0.90, the minimum required sample size was calculated as 56 participants, corresponding to at least 28 individuals in each group. The final sample size exceeded the minimum required number calculated by the a priori power analysis and was considered sufficient for the planned intergroup comparisons.

Comparisons between the buccally and palatally impacted canine groups were performed using independent-samples *t*-tests. All statistical tests were two-tailed, and the level of significance was set at *p* < 0.05. Effect sizes were calculated using Cohen’s d to quantify the magnitude of intergroup differences. Because the evaluated morphometric variables represented predefined and clinically related dimensions of maxillary arch morphology rather than exploratory outcomes generated post hoc, no formal adjustment for multiple comparisons was applied. Therefore, the findings should be interpreted in conjunction with the reported effect sizes and confidence intervals.

To assess measurement repeatability, 20 randomly selected individuals were re-evaluated by the same investigator after a two-week interval. Intraobserver reliability was assessed using the intraclass correlation coefficient (ICC) based on a two-way random-effects model with absolute agreement.

## 3. Results

A total of 86 individuals were included in the study, comprising 43 buccally impacted and 43 palatally impacted maxillary canine cases. The baseline characteristics of the study groups are presented in Table 1. No statistically significant differences were observed between the buccally and palatally impacted groups with respect to age (*p* = 0.518), sex distribution (*p* = 0.663), or side of impaction (*p* = 0.377). In addition, no significant differences were identified between the groups regarding malocclusion classification or skeletal pattern (*p* > 0.05), indicating that the groups were comparable with respect to the evaluated baseline characteristics.

Intraobserver reliability analysis demonstrated excellent agreement for all measurements. The ICC values ranged from 0.917 to 0.981, indicating high measurement reproducibility. Detailed ICC values for each measurement are presented in Table 2.

Statistically significant differences were observed between the groups for arch length (mean difference = 0.86; 95% CI: 0.06 to 1.66; *p* = 0.036; Cohen’s d = 0.46) and the arch length/arch width ratio (mean difference = 2.66; 95% CI: 0.63 to 4.69; *p* = 0.011; Cohen’s d = 0.56), both of which demonstrated higher mean values in the buccally impacted canine group. No statistically significant difference was found for arch width (mean difference = −0.68; 95% CI: −1.82 to 0.46; *p* = 0.233; Cohen’s d = −0.26) (Table 3).

Intermolar width was significantly greater in the palatally impacted canine group compared with the buccally impacted canine group (mean difference = −1.25; 95% CI: −2.17 to −0.33; *p* = 0.009; Cohen’s d = −0.57). No statistically significant differences were identified for palatal depth (mean difference = −0.28; 95% CI: −1.00 to 0.44; *p* = 0.443; Cohen’s d = −0.17) or the palatal depth/intermolar width ratio (mean difference = 0.86; 95% CI: −1.46 to 3.18; *p* = 0.460; Cohen’s d = 0.16) (Table 4).

Significant intergroup differences were identified for the four-incisor mesiodistal width (mean difference = 0.82; 95% CI: 0.11 to 1.53; *p* = 0.025; Cohen’s d = 0.49), available arch space (mean difference = −2.54; 95% CI: −4.14 to −0.94; *p* = 0.002; Cohen’s d = −0.68), and the four-incisor width/available arch space ratio (mean difference = 2.70; 95% CI: 1.76 to 3.64; *p* < 0.001; Cohen’s d = 1.23). The buccally impacted canine group demonstrated higher mean values for the four-incisor mesiodistal width and the corresponding ratio, whereas the palatally impacted canine group exhibited greater available arch space. The four-incisor width/available arch space ratio demonstrated the largest effect size among all evaluated variables (Table 5).

## 4. Discussion

The present study evaluated the relationship between the position of impacted maxillary canines and maxillary arch morphology using CBCT imaging and three-dimensional dental model analysis. The combined use of CBCT for accurate localization and classification of impacted canines and digital three-dimensional dental models for quantitative measurements enabled a comprehensive evaluation of maxillary arch morphology. This approach allowed standardized comparison of buccally and palatally impacted canines based on their confirmed three-dimensional position while ensuring reproducible morphometric assessment.

The present findings indicate that the position of impacted maxillary canines is associated with distinct morphological characteristics of the maxillary arch. Individuals with buccally impacted canines exhibited greater arch length and a higher arch length-to-arch width ratio, suggesting a predominance of sagittal arch development in this group. In contrast, the absence of a significant difference in transverse arch width between groups implies that sagittal components of arch morphology may play a more critical role in buccal impaction than transverse dimensions alone.

Previous studies examining the relationship between arch width and canine impaction have reported inconsistent results. While McConnell et al. proposed an association between palatal impaction and transverse maxillary deficiency, particularly in the anterior region [19], Al-Nimri and Gharaibeh suggested that increased transverse arch dimensions may contribute to the etiology of palatal canine impaction [26]. In agreement with the present findings, Chavez et al. reported no significant difference in transverse maxillary width between buccally and palatally impacted canines [25]. Similarly, several studies have found no significant differences in intermolar width between patients with palatally impacted or displaced canines and control groups [27,28,29,30].

Because isolated linear measurements may not fully capture the complex geometry of the maxillary arch, the arch length-to-arch width ratio multiplied by 100 was used to reflect the proportional relationship between sagittal and transverse dimensions. McNamara emphasized that simultaneous evaluation of these components is essential for understanding eruption patterns [31]. In this context, higher ratio values may represent a relatively elongated and narrow (triangular) arch morphology, in which sagittal dimensions predominate. Such a configuration may influence anterior tooth positioning and contribute to buccal displacement of the canines, consistent with previous reports identifying triangular arch forms more frequently in buccally impacted cases [25].

Conversely, Kim et al. reported higher arch length-to-intermolar width ratios in palatally impacted canines compared with buccally impacted cases [32]. These discrepancies likely reflect the multidimensional nature of arch morphology, as well as differences in sample characteristics and measurement methodologies, underscoring that arch geometry cannot be adequately represented by a single proportional parameter. Taken together, the present findings suggest that buccal canine impaction is associated with a morphological pattern characterized by sagittal arch elongation and relatively reduced dental arch width in relation to arch length rather than by absolute skeletal transverse deficiency.

Assessment of palatal morphology revealed significantly greater intermolar width in the palatally impacted canine group. Although arch width and intermolar width are conceptually related, they were measured using different anatomical reference points in this study. Arch width was defined using mesiobuccal cusp tips, whereas intermolar width was measured between mesiopalatal cusp tips. Variations in molar positioning, dentoalveolar compensation, and posterior tooth angulation may therefore explain the presence of significant differences in intermolar width despite comparable arch width values.

When interpreted alongside arch width measurements, the increased intermolar width observed in the palatally impacted group suggests that palatal impaction is not invariably associated with reduced dental arch width. This finding is consistent with previous reports indicating that palatal impaction may occur in individuals with varying dental arch morphologies [24]. The literature regarding palatal depth remains heterogeneous. While some studies have reported associations between palatal depth or palatal morphology and palatal canine impaction, others have demonstrated weak or inconsistent relationships [33]. Miresmaill et al. reported shallower palatal depth in palatally impacted canines [34], whereas Kim et al. observed reduced palatal depth in buccally impacted cases [32]. Similarly, Mehta et al. reported increased palatal depth in palatally impacted canines [24]. In contrast, a recent CBCT-based study found no significant difference in palatal depth between buccally and palatally impacted canine groups [22]. These contrasting findings further highlight the heterogeneity of the available evidence regarding the relationship between palatal morphology and impacted canine position.

In the present study, no significant intergroup differences were identified for palatal depth or the palatal depth–to–intermolar width ratio. These findings suggest that palatal depth alone may not be a decisive factor in determining the direction of canine impaction and that its interpretation should be integrated with transverse arch measurements.

Consistent with previous reports, buccally impacted canines were associated with greater indicators of tooth–arch size discrepancy. Recent CBCT-based evidence has demonstrated a significantly smaller maxillary arch perimeter on the impacted side in buccal impaction cases, further supporting the association of space-related dental arch characteristics with buccal canine impaction [22]. Similarly, previous findings have indicated that inadequate arch length, increased crowding, and reduced available arch space are more frequently associated with buccal canine impaction, whereas palatal impaction tends to be associated with reduced intermolar width and increased palatal depth [24]. The buccally impacted group demonstrated higher total mesiodistal width of the four incisors and a higher ratio of incisor width to available arch space, indicating a greater likelihood of anterior crowding and space deficiency [35].

The effect size analysis further emphasized the clinical relevance of these findings, as the ratio of four-incisor mesiodistal width to available arch space demonstrated a large effect size (Cohen’s d = 1.23), representing the strongest intergroup difference observed in this study. This finding suggests that tooth–arch size discrepancy may be an important characteristic associated with buccal canine impaction.

When considered together with increased arch length and higher arch length-to-arch width ratios, these findings indicate a morphological pattern characterized by sagittal elongation combined with relatively reduced dental arch width, which may represent a morphological characteristic associated with buccal canine impaction. Evaluation of available arch space alone also revealed lower values in the buccally impacted group, further supporting this interpretation. Mercuri et al. reported lower levels of crowding in patients with palatally impacted canines, suggesting greater available arch space in palatal cases and corroborating the present findings [36]. In contrast, Kim et al. found no significant difference in available arch space between buccally and palatally impacted groups [32].

Taken together, these findings suggest that the distinction between buccal and palatal canine impaction may be better characterized by overall patterns of maxillary dental arch morphology rather than by any single morphometric parameter alone. This observation may help explain some of the inconsistencies reported in previous studies that focused primarily on individual measurements. By evaluating arch morphology, palatal morphology, and tooth–arch size relationships within a single standardized three-dimensional analytical framework, the present study provides a more comprehensive characterization of the dentoalveolar patterns associated with different impaction positions.

This study has several limitations. The retrospective and cross-sectional design precluded longitudinal assessment of growth and eruption dynamics. Although the mean age of the sample was 16.1 ± 0.72 years, individual variations in craniofacial growth may still have influenced arch morphology. Additionally, the absence of a non-impacted control group limited direct comparison with normal maxillary arch morphology. Therefore, the present findings should be interpreted as differences between buccal and palatal impaction patterns rather than as evidence that either group deviates from normal maxillary arch morphology. Furthermore, the use of different anatomical reference points for specific measurements necessitates cautious interpretation of certain findings. Moreover, all morphometric measurements were derived from three-dimensional digital dental models and therefore primarily reflect dental arch morphology rather than skeletal or alveolar maxillary morphology. Although CBCT images were available for all participants, they were used exclusively for the localization and classification of impacted maxillary canines as buccal or palatal. Consequently, measurements such as arch width and intermolar width should be interpreted as indicators of dental arch characteristics rather than direct representations of skeletal maxillary width or alveolar morphology. Therefore, the present findings should not be interpreted as evidence of skeletal transverse maxillary deficiency. Another limitation is that interobserver reliability was not assessed for either the morphometric measurements or the CBCT-based buccal/palatal classification of impacted canines because all evaluations were performed by a single calibrated examiner.

Certain statistical considerations should also be acknowledged when interpreting the findings. Multiple intergroup comparisons were performed across several predefined morphometric variables. These variables were selected a priori because they represent related dimensions of maxillary arch morphology rather than independent exploratory outcomes. Nevertheless, no formal adjustment for multiple comparisons was applied. Although effect sizes and 95% confidence intervals were reported for all comparisons, the possibility of type I error cannot be completely excluded. Therefore, findings with smaller effect sizes and marginal *p* values should be interpreted with appropriate caution and confirmed in future studies. Furthermore, the a priori power analysis was based on a large anticipated effect size (Cohen’s d = 0.80). Therefore, the study may have been less sensitive to detecting small intergroup differences in some morphometric variables. Consequently, the absence of statistically significant differences for certain measurements should not necessarily be interpreted as evidence of complete equivalence between groups.

Future longitudinal studies including larger and more heterogeneous populations, as well as non-impacted control groups, are warranted to further clarify the relationship between maxillary arch morphology and impacted canine position. In addition, future investigations integrating skeletal, dentoalveolar, and growth-related parameters using standardized three-dimensional assessment protocols may provide a more comprehensive understanding of the etiological mechanisms underlying maxillary canine impaction.

Overall, the findings of the present study support the hypothesis that maxillary arch morphology differs according to the position of impacted maxillary canines and demonstrate that buccal and palatal impactions are associated with distinct dentoalveolar morphological patterns. From a clinical perspective, these findings emphasize the importance of comprehensive three-dimensional evaluation of sagittal and transverse arch dimensions together with tooth–arch relationships rather than reliance on isolated linear measurements alone. Early identification of these morphological characteristics may contribute to more individualized interceptive and orthodontic treatment strategies for impacted maxillary canines.

## 5. Conclusions

The findings of this study demonstrate that the position of impacted maxillary canines is associated with distinct maxillary arch morphological patterns. Buccally impacted canines were more frequently associated with sagittal arch elongation, increased arch length-to-arch width ratios, and greater tooth–arch size discrepancy, whereas palatally impacted canines were not consistently associated with reduced dental arch width or reduced available arch space.

These findings support the multifactorial nature of maxillary canine impaction and emphasize the importance of comprehensive three-dimensional evaluation of sagittal and transverse arch dimensions together with tooth–arch relationships. From a clinical perspective, three-dimensional assessment of maxillary arch morphology may provide additional information regarding the morphological characteristics associated with impacted maxillary canines and may assist clinicians in the morphological assessment of patients with impacted maxillary canines, thereby providing additional information for orthodontic assessment and treatment planning.

## Figures and Tables

**Table 1:** Descriptive Characteristics of the Study Sample.

Characteristic	Buccally Impacted (*n* = 43)	Palatally Impacted (*n* = 43)	*p*
**Female, ** ***n*** **(%)**	20 (46.5%)	22 (51.2%)	0.663
**Male, ** ***n*** **(%)**	23 (53.5%)	21 (48.8%)	
**Right side, ** ***n*** **(%)**	**28 (65.1%)**	**24 (55.8%)**	**0.377**
**Left side, ** ***n*** **(%)**	15 (34.9%)	19 (44.2%)	
**Age (years), Mean ± SD**	**16.05 ± 0.74**	**16.15 ± 0.70**	**0.518**

*n*: number of participants; SD: standard deviation. Sex distribution was compared using the chi-square test, and age comparison was performed using the independent-samples *t*-test. Statistical significance was set at *p* < 0.05.

**Table 2:** Intraobserver Reliability of Measurements.

Measurement	ICC
**Arch length**	0.927
**Arch width**	0.965
**Arch length/arch width ratio (%)**	0.918
**Palatal depth**	0.917
**Intermolar width**	0.954
**Palatal depth/intermolar width ratio (%)**	0.969
**Four-incisor mesiodistal width**	0.942
**Available arch space**	0.922
**Four-incisor width/available arch space ×100 (%)**	0.981

ICC: intraclass correlation coefficient. Intraobserver reliability was assessed using a two-way random-effects model with absolute agreement. ICC values greater than 0.90 indicated excellent measurement reliability.

**Table 3:** Comparison of Arch Length and Width Measurements Between Groups.

Measurement	Buccally Impacted (Mean ± SD)	Palatally Impacted (Mean ± SD)	Mean Difference (95% CI)	*p*	Cohen’s d
**Arch length**	36.16 ± 1.99	35.30 ± 1.73	0.86 (0.06 to 1.66)	**0.036**	0.46
**Arch width**	50.41 ± 3.07	51.09 ± 2.10	−0.68 (−1.82 to 0.46)	0.233	−0.26
**Arch length/arch width × 100**	71.89 ± 4.61	69.23 ± 4.90	2.66 (0.63 to 4.69)	**0.011**	0.56

Independent-samples *t*-test; mean differences are presented with 95% confidence intervals (CI); Cohen’s d was calculated as a measure of effect size; *p* < 0.05 was considered statistically significant.

**Table 4:** Comparison of Palatal Depth and Intermolar Width Measurements Between Groups.

Measurement	Buccally Impacted (Mean ± SD)	Palatally Impacted (Mean ± SD)	Mean Difference (95% CI)	*p*	Cohen’s d
**Palatal depth**	19.36 ± 1.45	19.64 ± 1.90	−0.28 (−1.00 to 0.44)	0.443	−0.17
**Intermolar width**	39.66 ± 2.25	40.91 ± 2.09	−1.25 (−2.17 to −0.33)	**0.009**	−0.57
**Palatal depth/intermolar width × 100**	49.01 ± 5.18	48.15 ± 5.52	0.86 (−1.46 to 3.18)	0.460	0.16

Independent-samples *t*-test; mean differences are presented with 95% confidence intervals (CI); Cohen’s d was calculated as a measure of effect size; *p* < 0.05 was considered statistically significant.

**Table 5:** Comparison of Four-Incisor Mesiodistal Width and Available Arch Space Measurements Between Groups.

Measurement	Buccally Impacted (Mean ± SD)	Palatally Impacted (Mean ± SD)	Mean Difference (95% CI)	*p*	Cohen’s d
**Four-incisor mesiodistal width**	27.82 ± 1.57	27.00 ± 1.75	0.82 (0.11 to 1.53)	**0.025**	0.49
**Available arch space**	69.73 ± 4.09	72.27 ± 3.38	−2.54 (−4.14 to −0.94)	**0.002**	−0.68
**Four-incisor width/available arch space ×100**	40.07 ± 2.48	37.37 ± 1.89	2.70 (1.76 to 3.64)	**<0.001**	1.23

Independent-samples *t*-test; mean differences are presented with 95% confidence intervals (CI); Cohen’s d was calculated as a measure of effect size; *p* < 0.05 was considered statistically significant.

## Data Availability

The data presented in this study are not publicly available due to ethical and privacy restrictions, as they are derived from anonymized patient radiographic records obtained from institutional archives. Data may be made available from the corresponding author upon reasonable request and with permission of the relevant ethics committee.

## Abbreviations

CBCT	Cone-beam computed tomography
3D	Three-dimensional
ICC	Intraclass correlation coefficient
SD	Standard deviation
